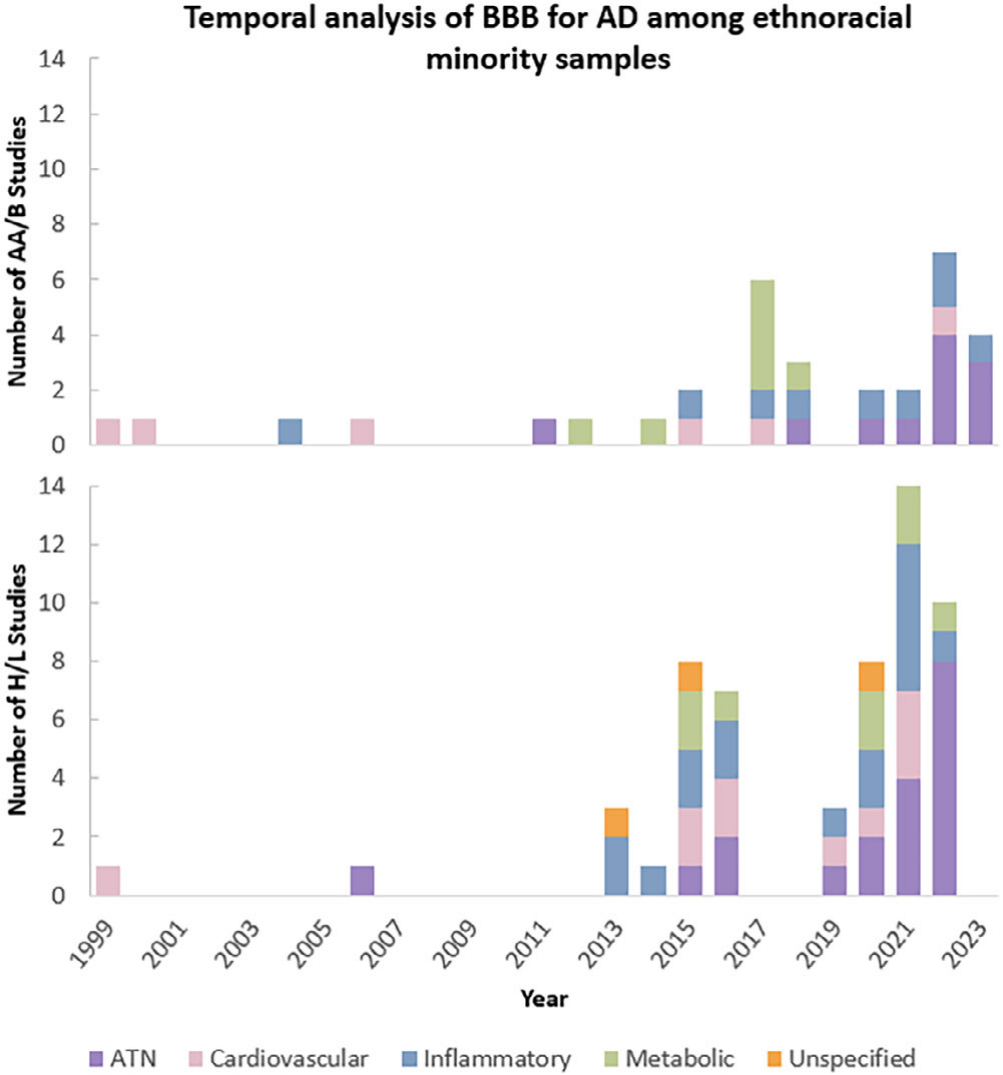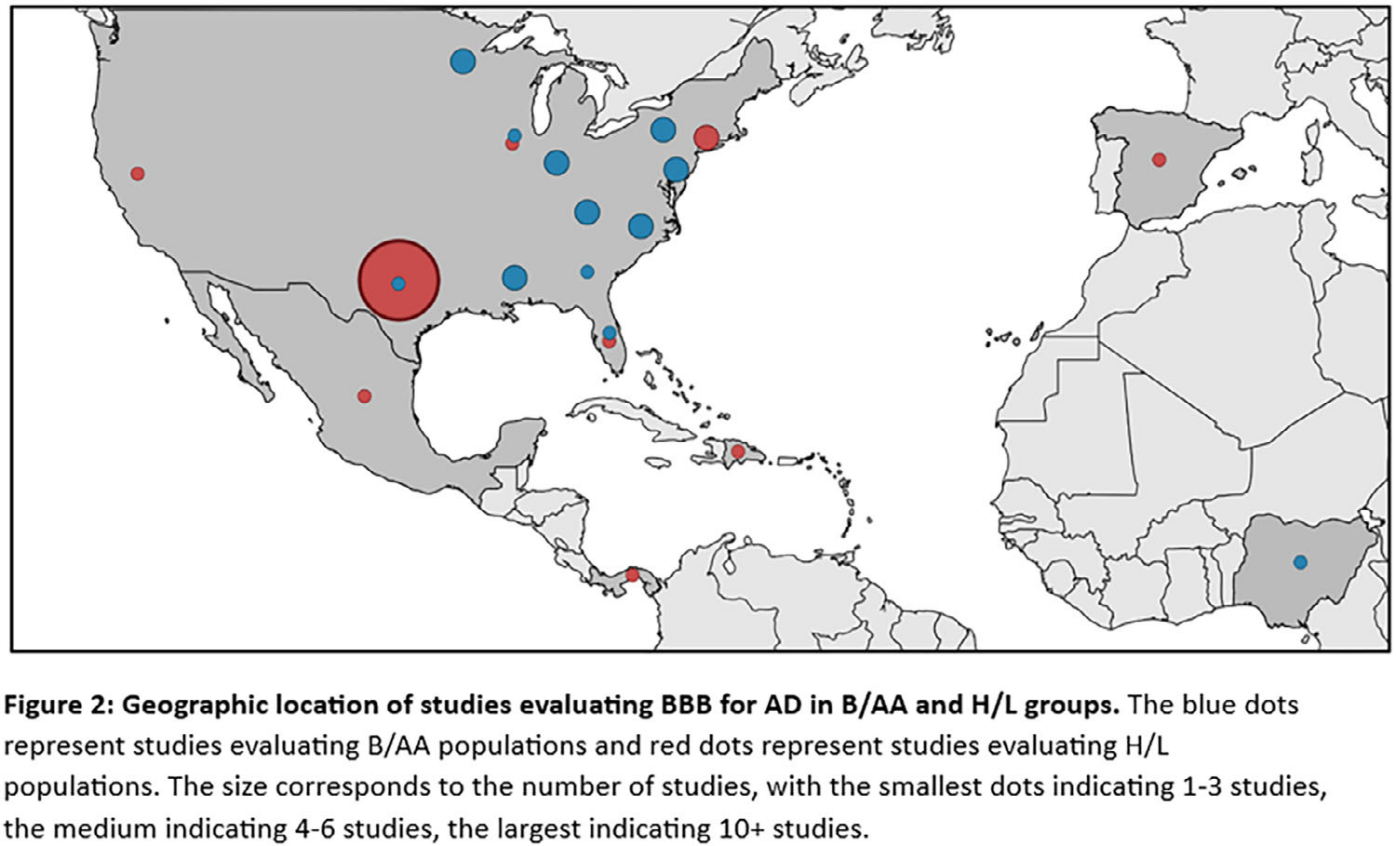# Blood‐based biomarkers for Alzheimer’s disease among ethnoracial minority samples – a scoping review

**DOI:** 10.1002/alz.085306

**Published:** 2025-01-09

**Authors:** Amanda Leisgang Osse, Amy Nguyen, Stacey Moeller, Jeffrey L. Cummings, Jefferson W Kinney, Samantha E John

**Affiliations:** ^1^ University of Nevada Las Vegas, Las Vegas, NV USA; ^2^ University of Nevada, Las Vegas, Las Vegas, NV USA; ^3^ Chambers‐Grundy Center for Transformative Neuroscience, Department of Brain Health, School of Integrated Health Sciences, University of Nevada Las Vegas, Las Vegas, NV USA

## Abstract

**Background:**

Approximately 6.7 million Americans, aged 65 and older, are living with Alzheimer’s disease (AD) dementia. Ethnoracial minority populations are at greater risk for AD. Black/African Americans (B/AA) are twice as likely than non‐Hispanic White (NHW) individuals to develop AD, and Hispanic/Latinos (H/L) are 1.5 times as likely. Accurate, timely, and accessible diagnosis of AD is limited. The use of blood‐based biomarkers (BBB) could improve treatment and care among ethnoracial groups at risk.

**Method:**

We performed a scoping review of the literature to summarize the current state of BBB research in B/AA and H/L populations. Searches through three databases (PubMed, APA PsycInfo, and Scopus) yielded 383 and 281 papers for B/AA and H/L populations, respectively. Papers were evaluated through title/abstract, followed by full paper review, and included in the final sample if plasma, serum, or whole blood biomarkers were collected in B/AA and H/L samples with pathological or cognitive evidence of AD etiology.

**Results:**

Overall, 19 B/AA (sample size range N=76‐5,228) and 30 H/L (sample size range N=51–3071) papers published between 1999 and June 1, 2023 met criteria. Publications evaluating ethnoracial minority samples increased over time and particularly in recent years (Figure 1). Studies included samples primarily from the United States, with majority of B/AA studies conducted in the eastern U.S., and majority of H/L studies conducted in Texas (Figure 2). We classified biomarkers into five categories, AD pathologies (amyloid, tau, neurodegeneration [ATN]), cardiovascular, inflammatory, metabolic, and unspecified. Studies evaluating ATN in both H/L and B/AA increased over time, with multiplex assays and Quanterix Simoa the most frequently used methods.

**Conclusion:**

There has been a progressive increase in investigations of BBB for AD across all ethnoracial groups. Consideration of the blood sample measured, demographics of the individuals included, and location of the studies is required. Standardization of methods used and biomarkers evaluated, as well as replication of studies across larger and broader samples are needed to allow reliable conclusions to be drawn. Better understanding of BBB of AD in H/L and B/AA populations would advance the diagnosis and treatment of AD in these at‐risk ethnoracial groups.